# Alectinib and SALL4-Targeted Fatty Acid Oxidation: A Strategy to Combat Oxaliplatin Resistance in Gastric Cancer

**DOI:** 10.5152/tjg.2025.24495

**Published:** 2025-06-23

**Authors:** Yangbin Xiao, Kaining Fang, Jian Liao, Houwu Zhou, Weidong Zhu, Zheng Liu, Hui Ouyang, Ke Liu

**Affiliations:** 1 Department of Gastrointestinal Surgery, Yueyang People’s Hospital, Yueyang, China; 2 Department of Gynecology, Yueyang Maternal and Child Health Care Hospital, Yueyang, China; 3 Department of Gastrointestinal Surgery, the First Hospital of Changsha, Changsha, China; 4 Department of Hepatobiliary and Pancreatic Surgery, Yueyang People’s Hospital, Yueyang, China

**Keywords:** Alectinib, fatty acid oxidation, GC, oxaliplatin resistance, SALL4

## Abstract

**Background/Aims:**

Oxaliplatin is a frontline chemotherapeutic agent for gastric cancer (GC) patients; yet, its clinical efficacy is often hindered by drug resistance. Recent studies have suggested a link between fatty acid oxidation (FAO) in GC and chemoresistance, but the precise mechanisms remain elusive.

**Materials and Methods:**

In this study, *SALL4* was identified as a gene that is not only overexpressed in GC but also remarkably enriched in the FAO pathway through differential gene expression screening and gene set enrichment analysis. *SALL4* could enhance the FAO process and oxaliplatin resistance in GC, as corroborated by western blot, assessment of FAO rates and adenosine triphosphate levels, and cell counting kit-8.

**Results:**

Reversal experiments demonstrated that the small molecule drug Alectinib can counteract the promotion of FAO and oxaliplatin resistance by the upregulation of *SALL4*. The binding relationship between Alectinib and SALL4 protein was validated through molecular docking simulations and cellular thermal shift assay.

**Conclusion:**

This research has brought to light that Alectinib targets *SALL4* to modulate the FAO process, thereby reducing the oxaliplatin resistance of GC cells. These findings may open up new avenues to tackle chemoresistance in GC.

**Main Points**

- High expression of SALL4 mediates fatty acid oxidation (FAO) and enhances chemotherapy resistance of gastric cancer (GC) cells.
- Alectinib has a stable affinity for the SALL4 protein.
- Alectinib interacts with SALL4 to inhibit FAO and chemoresistance in GC cells.

## Introduction

Statistics on gastric cancer (GC) are sobering, showing a high mortality rate of 75% in most regions around the world and a 5-year survival rate for advanced cases that is less than 30%, positioning it as the third most common cause of cancer-related deaths.^1^^,^^2^ The youth are not immune, with the growing prevalence of obesity and gastroesophageal reflux disease heightening their vulnerability to GC.^3^ The demographic trends of population expansion and aging are only to exacerbate the situation, bringing more GC cases in the future. Early detection offers a chance for surgical cure, but this window often closes as most GC patients present at advanced stages. The therapeutic options for advanced GC encompass radiotherapy, chemotherapy, molecular-targeted therapies, and immunotherapy.^4^^,^^5^ Beyond the complexity of tumor heterogeneity and the tardiness of diagnosis, drug resistance is a major obstacle in the clinical management of GC. Unraveling the mechanisms of GC drug resistance is necessary to improve patient treatment and prognostic outcomes.

Oxaliplatin, the third-generation platinum drug, mainly acts on DNA to prevent its replication and transcription. The chronic use of drugs like oxaliplatin can lead to the evolution of resistance in cancer cells, which compromises the success of chemotherapy.^6^ It has been discovered that metabolic disorders, which are indicative of cancer development, may also be connected to chemoresistance.^7^ Fatty acid oxidation (FAO) stands out as the principal route for the metabolism of fatty acids and the concomitant production of adenosine triphosphate (ATP) and nicotinamide adenine dinucleotide phosphate. Wang et al^8^ have reported in breast cancer that the JAK/STAT3 signaling can facilitate the transcription of CPT1B, thereby promoting FAO and sustaining the proliferation of breast cancer stem cells as well as their chemoresistance. Zhu et al^9^ found that in epithelial ovarian cancer, the depletion of NKX2-8 resulted in the reprogramming of FAO in an adipose microenvironment, leading to cisplatin resistance. These studies illuminate the correlation between FAO and chemoresistance in GC cancer, and in-depth discussion is required on the mechanisms by which lipid metabolism could dictate cancer cell fate and regulate resistance to therapy.

*SALL4*, the human homolog of the Drosophila Spalt gene, exists in 2 isoforms, SALL4A and SALL4B, and is situated on chromosome 20q13.2.^9^ It is instrumental in the upkeep of embryonic stem cell pluripotency and self-renewal. SALL4 expression is typically silent in adult tissues, with the exception of germ cells and hematopoietic stem cells.^10^ However, its re-expression is a common feature in various solid tumors and hematological malignancies, such as lung cancer,^11^ hepatocellular carcinoma,^12^ and colorectal cancer.^13^ SALL4, when overexpressed in GC tissues, signals a worsened prognosis for GC patients. Evidence shows that when SALL4 levels are high, the Wnt/β-catenin signaling pathway will be triggered, potentially due to the upregulation of the SALL4 co-expressed gene TRIB3, which is linked to the genesis of GC.^14^ Moreover, SALL4 accelerated GC progression by regulating hexokinase 2 (HK-2) and enhancing glycolysis.^15^ These findings encourage the exploration of SALL4 as a potential therapeutic target for GC diagnosis and treatment, prompting further investigation into its carcinogenic role and potential as a therapeutic target, as well as the development of drugs to target this transcription factor (TF).

Our investigation, grounded in molecular and cellular experimentation, exposes the elevated expression of SALL4 in GC and its role in fostering resistance to oxaliplatin. By combining bioinformatics with rescue experiments, a connection was found between SALL4 and the metabolic dysregulation in GC. Through molecular docking simulations, a small molecule drug (Alectinib) that hones in on SALL4 was also identified. This research illustrates that Alectinib, by engaging SALL4, inhibits FAO and mitigates the resistance of GC cells to oxaliplatin, offering a strategy to ameliorate drug resistance in GC patients.

## Materials and Methods

### Bioinformatics Analysis

Data on GC mRNA expression levels (normal: 32, tumor: 375) were downloaded from The Cancer Genome Atlas (TCGA) database (https://portal.gdc.cancer.gov/). Our differential expression analysis between normal and tumor mRNA groups was facilitated by the *edgeR* package, identifying mRNAs with significant differences based on the criteria of |logFC| > 2 and FDR < 0.05. The gene *SALL4* was subsequently chosen for further study following a literature review. A* t*-test was conducted to evaluate the expression of *SALL4* in GC versus normal tissues using TCGA-GC patient data. GSEA was then applied to conduct a pathway enrichment analysis for *SALL4*, which was complemented by a Pearson correlation analysis to assess the relationship between *SALL4* and genes within the enriched pathways.

### Molecular Docking Simulation

The RCSB-PDB database (https://www.rcsb.org/) was the source for downloading the SALL4 protein structure, which underwent preprocessing in PyMOL to eliminate any redundancy and add hydrogen atoms. The prediction of the receptor’s pocket structure was facilitated by the ProteinsPlus (https://proteins.plus/) online analysis tool. Small molecule ligands in mol2 format were extracted from the Zinc database (https://zinc.docking.org/substances/subsets/fda/), split into individual mol2 files with OpenBabel, and converted into pdbqt format using MGLTools. The docking of small molecules into the protein pocket was simulated with AutoDock Vina, and the minimum binding energy data were saved. The interactions between the small molecules and surrounding residues were depicted in 3D and 2D using PyMOL and Ligplot software.

### Cell Cultivation

The cell lines GES-1 (SNL-304), a human gastric mucosal cell line, and the human GC cell lines AGS (SNL-103), HGC-27 (SNL-104), and MKN-74 (SNL-176) were sourced from SUNNCELL (China). The selection of tumor cells was based on previous studies related to GC.^16^^,17^ Additionally, there are differences in the origins of the 3 cell lines (https://www.cellosaurus.org/). The oxaliplatin-resistant variant HGC-27/L (MXC850) was supplied by Shanghai MEIXUAN Biological Science and Technology Co., Ltd. (China). The GES-1, HGC-27, HGC-27/L, and MKN-74 cells were maintained in RPMI-1640 medium (Procell, China) with 10% FBS and 1% P/S. The AGS cells were cultivated in F12K medium (Procell, China) with 10% FBS and 1% P/S, all under conditions of 37°C and 5% CO_2_ in a humidified atmosphere. Patient informed consent is not applicable in this study. Ethical approval is not required for this study.

### Cell Transfection

HGC-27/L cells were seeded in 6-well plates to 50% confluence and allowed to incubate overnight. *SALL4* was overexpressed by inserting its coding sequence (CDS) into the pcDNA3.1 expression vector (Invitrogen, USA), with the empty vector as a negative control. *SALL4* siRNA and its scramble control were chemically synthesized and obtained from Genechem (China). Transfection of the above plasmids or oligonucleotides into cells was performed using the LipoFiter reagent (Hanbio, China) in a serum-free environment. After 6 hours of transfection, the cells were cultured in a complete growth medium for 42 hours.

### Quantitative Reverse Transcription Polymerase Chain Reaction

The TRIzol reagent (Invitrogen, USA) was applied to extract total RNA from cells. This RNA was reverse transcribed into cDNA using the Reverse Transcription Master Mix for Quantitative polymerase chain reaction (qPCR) II (MCE, USA). Subsequent amplification of the cDNA was conducted on a 7500 Fast Real-time PCR System (Applied Biosystems, USA) with the aid of SYBR Green qPCR Master Mix (MCE, USA). The relative expression of *SALL4* was quantified relative to β-actin, using the 2^−ΔΔCt^ method. The results from 3 sets of replicate experiments were integrated, and the differences between each group and its control group were analyzed using Student’s *t*-test. Primer specifics are detailed in Table 1.

### Cell Counting Kit - 8

The CCK-8 assay kit (Elabscience, China) was implemented to gauge cell viability. HGC-27/L cells were seeded at 5 × 10^3^ cells per well in a 96-well plate and were treated with a spectrum of oxaliplatin concentrations (0, 2.5, 5, 10, 20, 40, 80 μg/mL). After 48 hours, 10 μL of CCK-8 solution was introduced into each well for another 2-hour incubation. The microplate reader (PerkinElmer, USA) measured the absorbance at 450 nm. A dose-response curve was plotted to represent the drug concentration versus the cell viability percentage, and the IC_50_ values (50% growth suppression) were derived for each cell group. The results of the 3 sets of replicates were integrated, and the differences between each group and its control group were analyzed using Student’s *t*-test.

### Western Blot

Total protein was extracted with radio immunoprecipitation assay buffer (CST, USA) fortified with protease and phosphatase inhibitors. The BCA Protein Assay Kit (Sigma-Aldrich, USA) was applied for protein quantification. Equal volumes of protein samples were run on a 10% SDS-PAGE and transferred onto nitrocellulose membranes (Bio-Rad, USA). The membranes were blocked with 5% milk for 1 hour, then incubated with primary antibodies at 4°C overnight, including CPT1 (15184-1-AP, Proteintech, USA), ACS (ab133664, Abcam, UK), FATP (ab81875, Abcam, UK), SALL4 (ab29112, Abcam, UK), and β-actin (ab8227, Abcam, UK). The cells were washed and incubated with the secondary antibody, goat anti-rabbit IgG H&L (HRP) (ab205718, Abcam, UK), for 2 hours at room temperature. Finally, detection was carried out with an Enhanced Chemiluminescence (ECL) reagent (Thermo Fisher Scientific, USA).

### Fatty Acid Oxidation and Adenosine Triphosphate Detection

Mitochondria were purified from cells using the Cell Mitochondria Isolation Kit (C3601, Beyotime, China). The mitochondrial protein concentration was measured before the FAO rate was determined with the FAO Assay Kit (BR00001, Assaygenie, Ireland). Cells were harvested, lysed, and subjected to centrifugation at 12 000 g for 10 minutes at 4°C. The supernatant was then assessed for ATP levels using the ATP Content Colorimetric Assay Kit (E-BC-K157-M, Elabscience, China). The results from 3 sets of replicate experiments were integrated, and the differences between each group and its control group were analyzed using Student’s *t*-test.

### Cellular Thermal Shift Assay

Following 9 hours of incubation with or without Alectinib, cells were harvested for CETSA. Briefly, the cells were divided into 6 equal parts and heated at various temperatures (44, 48, 52, 56, 60, and 64°C) for 3 minutes each. The heated cells were then kept at −80°C for 12 hours, and allowed to thaw at room temperature aqueous solution for 5 minutes, and this process was repeated to ensure complete fragmentation of the cells. Cell lysates were extracted by centrifugation at 20 000 g for 20 minutes, and SALL4 levels were analyzed by western blot.

### Statistical Analysis

Data analysis was conducted utilizing GraphPad Prism8.0 (GraphPad software; La Jolla, USA). Each experiment was repeated at least 3 times, and data were displayed as “mean ± SD.” The Student’s *t*-test assessed variations between 2 groups, adopting a 95% CI, with significance set at *P *< .05.

## Results

### SALL4 Upregulation in Gastric Cancer Enhances Oxaliplatin Resistance

**Figure 1.**
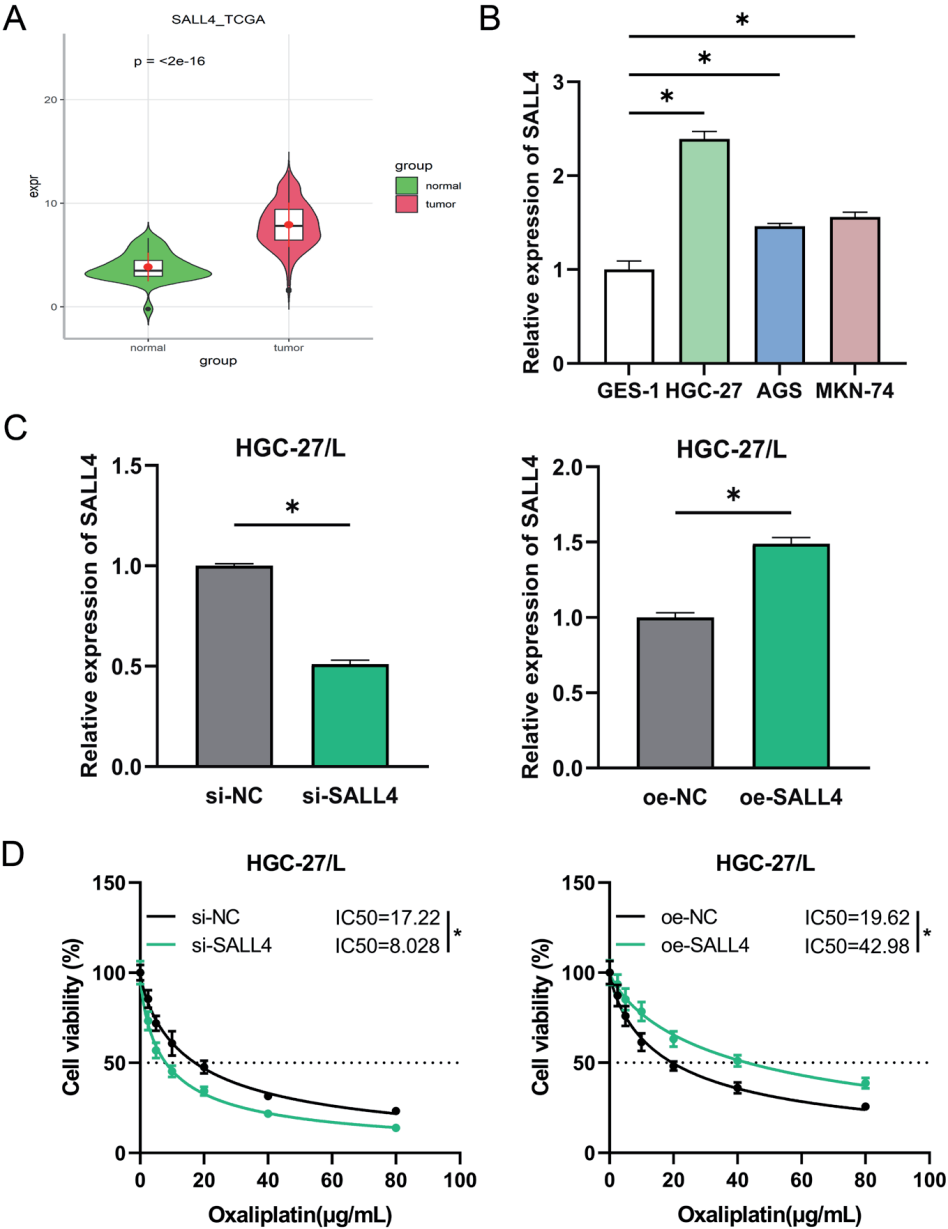
SALL4 Upregulation in GC Enhances Oxaliplatin Resistance. A: Analysis of *SALL4* expression in tumor samples in contrast to normal samples using the TCGA dataset; B: qRT-PCR determined *SALL4* mRNA levels in gastric mucosal cells (GES-1) and GC cell lines (HGC-27, AGS, MKN-74); C: qRT-PCR assessed transfection efficiency for si-NC, si-SALL4, oe-NC, oe-SALL4 groups; D: CCK-8 gauged the inhibitory effect of oxaliplatin on HGC-27/L cell viability, with the IC_50_ values calculated. * represents *P *< .05.

Differential mRNA expression analysis of GC patient data from TCGA identified 1677 differentially expressed mRNAs (DEmRNAs), with 897 genes upregulated and 781 downregulated (Supplementary Table 1). A literature review led to the selection of *SALL4* as the principal gene for this study.* T*-test analysis within the TCGA-GC dataset revealed substantial *SALL4* overexpression in cancer tissues as opposed to normal tissues (Figure 1A). Quantitative reverse transcription polymerase chain reaction also detected higher* SALL4* mRNA levels in GC cell lines (HGC-27, AGS, MKN-74) when contrasted with the non-cancerous gastric mucosal cell line (GES-1) (Figure 1B). *SALL4* showed the highest relative expression in HGC-27 cells. Therefore, further research using HGC-27 cells was conducted.

Existing research has indicated that SALL4 expression is correlated with chemoresistance in breast and lung cancers.^18^^,^^19^ To explore the impact of SALL4 on oxaliplatin resistance in GC, the oxaliplatin-resistant strain (HGC-27/L) was obtained and established si-NC/si-SALL4/oe-NC/oe-SALL4 cell groups. After evaluating transfection efficiency (Figure 1C), the CCK-8 assay kit was used to calculate the IC_50_ values for cells in each group following oxaliplatin treatment (Figure 1D). The results indicated a marked decrease in the IC_50 _value for the si-SALL4 group when compared to the si-NC group, while the oe-SALL4 group had a higher IC_50 _value than the oe-NC group. These findings indicate that high SALL4 expression in GC may enhance resistance to oxaliplatin.

### SALL4 Drives Fatty Acid Oxidation and Oxaliplatin Resistance in Gastric Cancer

**Figure 2.**
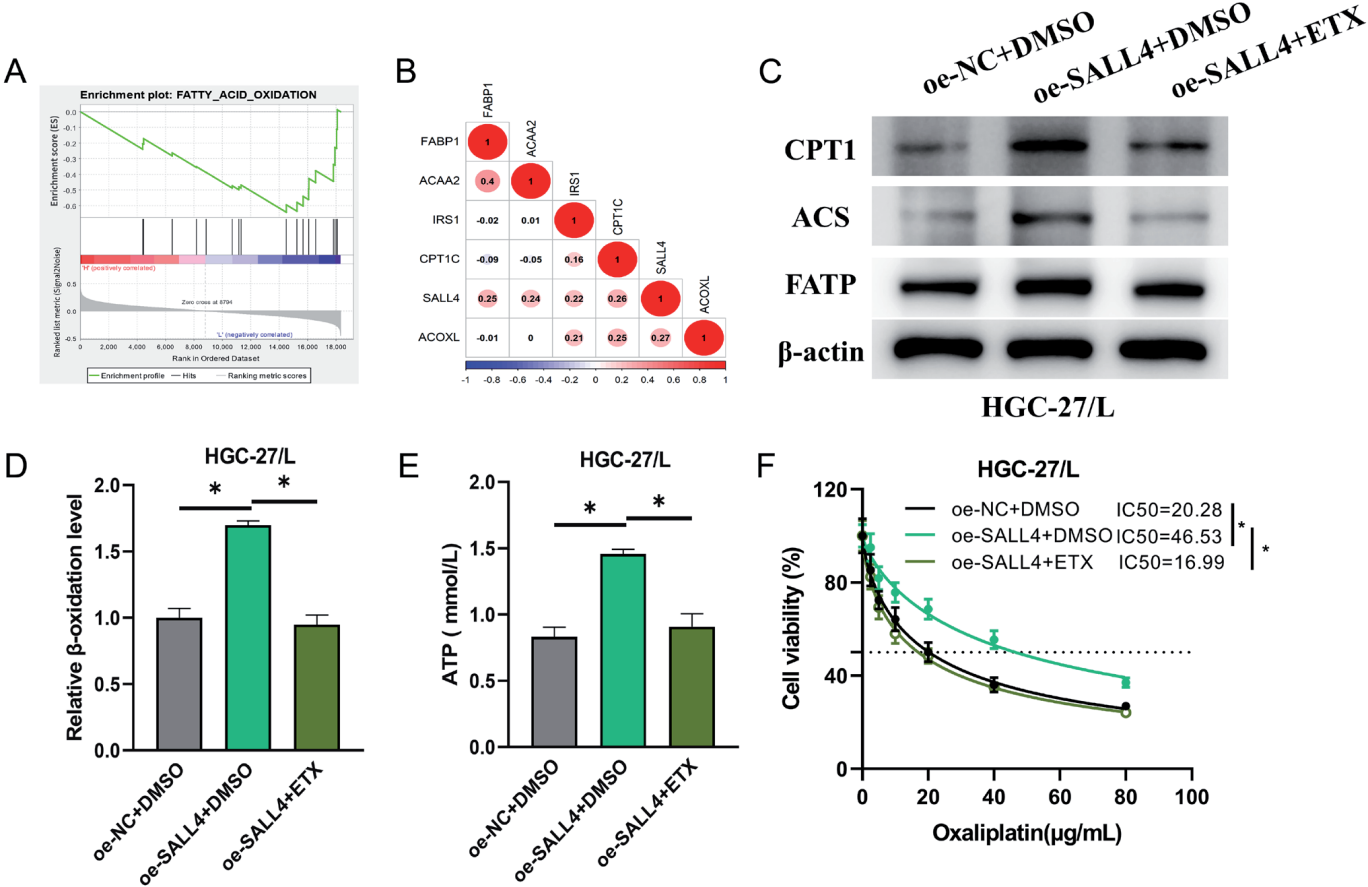
SALL4 Drives FAO and Oxaliplatin Resistance in GC. A: GSEA plot for SALL4 in the FAO pathway; B: Correlation analysis of SALL4 with central FAO genes (ACOXL, CPT1C, FABP1, ACAA2, IRS1); C: WB analysis of FAO-associated protein levels (CPT1, ACS, FATP) among different treatment groups; D: FAO rate measurement with a colorimetric assay kit for fatty acid β-oxidation; E: ATP levels evaluation using an ATP detection kit; F: Determination of IC_50_ value for the effect of oxaliplatin on HGC-27/L cell survival via the CCK-8 assay kit. * represents *P *< .05.

To delve into the underlying reasons for SALL4’s impact on oxaliplatin resistance in GC, GSEA enrichment analysis was undertaken, which showed notable enrichment of SALL4 in the FAO pathway (Figure 2A). A correlation analysis highlighted a positive association between SALL4 and key FAO pathway genes (ACOXL, CPT1C, FABP1, ACAA2, IRS1) (Figure 2B). The HGC-27/L cell line was used to establish the following groups: oe-NC+DMSO, oe-SALL4+DMSO, and oe-SALL4+etomoxir (ETX) to scrutinize the influence of SALL4-mediated FAO on GC cell resistance to oxaliplatin. The levels of FAO-associated proteins (CPT1, ACS, FATP), the rate of fatty acid β-oxidation, and ATP levels were elevated with SALL4 overexpression but normalized with the addition of ETX (Figure 2C-E). The CCK-8 assay, used for assessing cell viability post-oxaliplatin treatment, showed an impressive increase in the IC_50 _value for the oe-SALL4+DMSO group, which was notably reduced with the concurrent use of the FAO inhibitor (Figure 2F). These findings suggest that SALL4 may promote FAO, thereby increasing the resistance of GC cells to oxaliplatin.

### Alectinib Binds to SALL4

**Figure 3.**
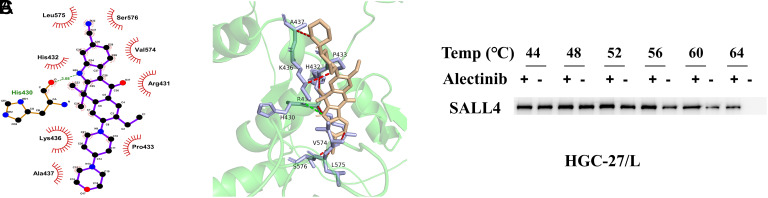
Alectinib Binds to SALL4. A-B: 2D and 3D visualizations of the hydrogen bonds and hydrophobic interactions between Alectinib and SALL4; C: CETSA-WB assay substantiating the binding interaction between Alectinib and SALL4.

In an attempt to reverse the negative influence of SALL4 in GC, small molecule drugs were screened that can target the SALL4 protein. Through molecular docking simulations, Alectinib was identified for its strong binding affinity and low energy interaction with SALL4, prompting an examination of the potential binding modes. Two-dimensional and 3D diagrams illustrating the hydrogen bonding and hydrophobic interactions between Alectinib and SALL4 (Figure 3A-B) showed that residues Leu575, Ser576, His432, Val574, Arg431, Lys436, Pro433, and Ala437 of Alectinib interacted hydrophobically, forming a set of hydrogen bonds with residue His430. CETSA-WB assays showed that the SALL4 protein was prone to degradation as temperature rises, yet when complexed with Alectinib, more SALL4 remains intact at the same temperatures, evidencing stable binding between Alectinib and SALL4 (Figure 3C).

### Alectinib Targets SALL4 to Mediate Fatty Acid Oxidation and Reduce Chemoresistance in Gastric Cancer Cells

**Figure 4.**
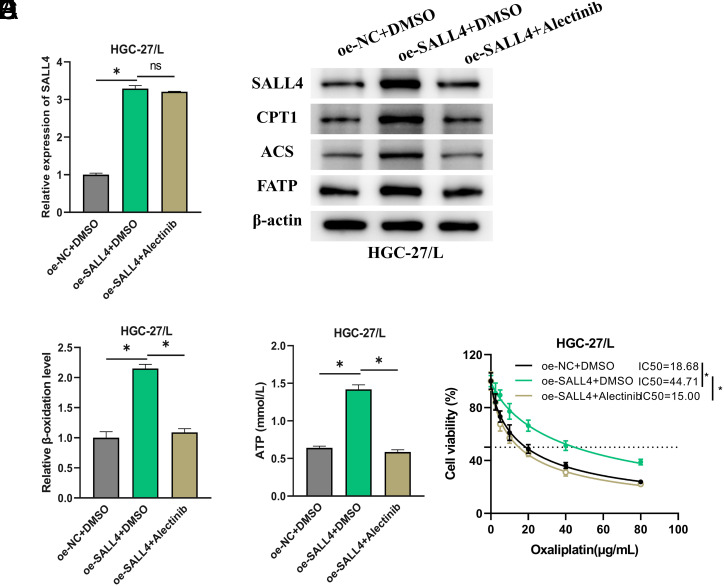
Alectinib Targets SALL4 to Mediate FAO and Reduce Chemoresistance in GC Cells. A: Quantitative reverse transcription polymerase chain reaction analysis of *SALL4* mRNA expression in cells; B: Western Blot detection of SALL4 and FAO-related protein (CPT1, ACS, FATP) expression; C: FAO rate measured by a colorimetric assay kit for fatty acid β-oxidation; D: ATP levels assessed by an ATP assay kit; E: CCK-8 assay kit for evaluating the viability of HGC-27/L cells and calculating the IC_50_ value. * represents *P *< .05.

Continuing the investigation, rescue experiments were performed to determine the effects of Alectinib binding to SALL4 on FAO and oxaliplatin resistance. Three HGC-27/L cell groups were developed: oe-NC+DMSO, oe-SALL4+DMSO, and oe-SALL4+Alectinib. Quantitative reverse transcription polymerase chain reaction results demonstrated a marked increase in SALL4 mRNA levels in the last 2 groups relative to the control, signifying effective plasmid transfection, and showed no critical impact of Alectinib on *SALL4* mRNA expression (Figure 4A). WB detected that oe-SALL4 transfection enhanced SALL4 protein levels in cells, along with an increase in FAO-related proteins (CPT1, ACS, FATP); however, Alectinib treatment led to a reduction in SALL4 protein levels and a decrease in FAO-related proteins (Figure 4B). Similar patterns were noted in the fatty acid β-oxidation rate, ATP levels, and IC_50_ value of HGC-27/L cells (Figures 4C-E). Overall, Alectinib targets SALL4, thereby reducing FAO levels and suppressing the resistance of GC cells to oxaliplatin.

## Discussion

Oxaliplatin-based chemotherapy offers a lifeline to many GC patients, but the development of drug resistance has greatly impeded the efficacy of such treatments. It is essential to clarify the mechanisms of oxaliplatin resistance and identify new therapeutic targets to increase the effectiveness of chemotherapy, thus improving patient outcomes. This study discovered that the *SALL4* gene, overexpressed in GC, is markedly enriched in the FAO pathway, with a positive correlation to genes that promote FAO as indicated by correlation analysis. Integrating bioinformatics and experimental validation, it has been shown that SALL4 enhances oxaliplatin resistance in GC cells by mediating FAO, an effect that can be mitigated by the small molecule Alectinib.

SALL4, a TF, through transcriptional mechanisms, is able to regulate signaling pathways or oncoproteins that advance tumor progression, such as Wnt/β-catenin^20^ and the apoptotic proteins Bcl-2 and Bax.^21^ A meta-analysis has linked the activation of SALL4 to an elevated risk of cancer-related mortality and recurrence, with SALL4-expressing patients experiencing a critical rise in overall death rates and disease relapse, underscoring that SALL4 is associated with reduced survival and serves as a potential biomarker for cancer prognosis.^22^ Our study initially investigated the potential effects of SALL4 on GC, indicating its abnormal overexpression and its role in fostering resistance to oxaliplatin (evidenced by higher IC_50_ values). This finding is consistent with conclusions drawn in previous studies on breast cancer,^18^ lung cancer,^23^ and colorectal cancer.^24^ It is often observed that SALL4 is highly expressed in drug-resistant cell lines, and downregulating SALL4 can restore the chemotherapy sensitivity of tumor cells. These studies collectively support the role of SALL4 in promoting chemotherapy resistance in tumors, despite variations in the chemotherapy drugs and mechanisms discussed in each study.

In the research of cancer cell metabolism, the emphasis has been skewed toward glucose metabolism, overshadowing the fact that fatty acids, on a per-gram basis, are more energy-dense than glucose. In fact, lipid metabolism is a signature of cancer. In many aggressive cancers, there is an overabundance of FAO-related proteins, and irregular FAO activity has been linked to diverse facets of tumor progression,^25^ including proliferation, metastasis, cell survival, stem cell characteristics, and chemoresistance. Wang et al^26^ conducted a clinical analysis in gastrointestinal cancer, revealing that the high expression of key FAO enzymes is linked to unsatisfactory results from oxaliplatin-based chemotherapy; furthermore, they have confirmed that fatty acid catabolism is turned on in cells treated with oxaliplatin, which exhibit a higher expression of CPT1B and CPT2, and that the use of perifosine to block CPT-mediated FAO, when combined with oxaliplatin treatment, proves effective in preventing the advancement of gastrointestinal cancer. This study conducted GSEA enrichment and correlation analysis to reveal that the SALL4 gene is highly enriched in the FAO pathway, correlating positively with genes that promote FAO. Overexpression of SALL4 (oe-SALL4) markedly heightened the expression of FAO-associated proteins (CPT1, ACS, FATP), the rate of fatty acid β-oxidation, and ATP levels. The IC50 values confirmed that the activation of FAO intensifies the resistance of oxaliplatin-resistant GC cells (HGC-27/L) to the medication. In addition, ETX was observed to reverse the aforementioned effects induced by FAO. This result is corroborated by the study conducted by Chen et al.^27^ Based on this, it was speculated that SALL4 likely functions as a classical TF,^28^^,^^29^ activating the expression of FAO-related proteins by binding to the promoters of key genes. This, in turn, potentially enhances tumor cell resistance by adjusting the overall metabolic levels.

To neutralize the detrimental actions of SALL4 in GC, Alectinib, a small molecule drug that targets SALL4, was identified through molecular docking simulations. Cellular thermal shift assay–WB experiments unearthed the stable binding of Alectinib to the SALL4 protein. Rescue experiments validated that Alectinib could target SALL4 to decrease the resistance of GC cells to oxaliplatin by downregulating FAO levels. Alectinib is recognized as an ATP-competitive small molecule and a second-generation ALK inhibitor.^30^ Wu et al^31^ found that Alectinib markedly improved disease-free survival rates in ALK-positive non-small cell lung cancer patients after surgery when used as an adjuvant, outperforming platinum-based chemotherapy. Alectinib, a lipophilic basic compound, is known for excellent permeability, minimal liver metabolism-induced systemic clearance, extensive distribution, and moderate bioavailability.^32^ However, existing clinical trials have indicated that treatment with Alectinib may lead to adverse reactions in over 10% of patients, including varying degrees of constipation, nasopharyngitis, anemia, or peripheral edema. Some patients have even discontinued treatment due to abnormal liver function and interstitial lung disease.^33^^,^^34^ Therefore, the use of this drug in GC warrants more stringent safety confirmation.

This study elucidates the molecular mechanism whereby Alectinib targets SALL4 to mediate FAO, suppressing the resistance of GC cells to oxaliplatin. The combined use of Alectinib with chemotherapy drugs may provide a new avenue for overcoming chemoresistance in GC. However, the experimental design of this study has limitations: first, no animal models were included to verify the impact of Alectinib/SALL4 on in vivoFAO levels and the efficacy of oxaliplatin treatment; second, the molecular mechanism of SALL4 mediated FAO remains to be further explored. Altogether, this work suggests a feasible strategy for overcoming chemoresistance in GC and improving clinical treatment modalities, paving the way for the advancement and personalization of anti-cancer drug regimens.

## Supplementary Materials

Supplementary Material

## Figures and Tables

**Table 1.** Quantitative Reverse Transcription Polymerase Chain Reaction Primers

**Gene**	**Primer Sequence**
*SALL4*	forward primer 5’-TCGATGGCCAACTTCCTTC-3’
	reverse primer 5’-GAGCGGACTCACACTGGAGA-3’
*β-actin*	forward primer 5’-CACGAAACTACCTTCAACTCC-3’
	reverse primer 5’-CATACTCCTGCTTGCTGATC-3’

## Data Availability

The data that support the findings of this study are available on request from the corresponding author.
