# Supplementary material for: Alectinib and SALL4-Targeted Fatty Acid Oxidation: A Strategy to Combat Oxaliplatin Resistance in Gastric Cancer
Source: Turk J Gastroenterol. 2025 Jun 23;36(12):813–21. doi: 10.5152/tjg.2025.24495 (PMC12684282; doi:10.5152/tjg.2025.24495)
Supplement: Supplementary Material [file supplementary_material.pdf]

Supplementary Table 1. Differential analysis of mRNA expression data in GC identified 1677 DEmRNAs.

https://docs.google.com/document/d/1ynvoVvUDmecjl02jCjA-XcMXCkRz7G7FZwi4tpynOQE/edit?usp=sharing
